# The power of digital story in early mathematics education: Innovative approaches for children with intellectual disabilities

**DOI:** 10.1371/journal.pone.0302128

**Published:** 2024-04-16

**Authors:** Özlem Altindağ Kumaş

**Affiliations:** Department of Special Education, Faculty of Ziya Gökalp Education, Dicle University, Diyarbakır, Turkey; Durham University, UNITED KINGDOM

## Abstract

This study explored the effectiveness of digital story interventions in improving early math skills in kindergarten children with mild intellectual disabilities. Digital stories are multimedia narratives that combine text, images, and audio to enhance learning experience. This experimental study used a pretest-posttest control group design. The intervention group consisted of 15 children who participated in an 8-week digital story intervention targeting early math skills. A matched control group was used to control for sex differences. Data were collected through the TEMA-3 test scores and teacher and child feedback. Post-intervention, the experimental group showed significant improvements in TEMA-3 test scores compared to the control group. Teachers and children reported a positive perception of the intervention’s social validity, highlighting enhanced engagement and understanding of math concepts. This study demonstrated that digital story-based education is a promising approach for improving early math skills in children with mild intellectual disabilities. These findings suggest potential implications for integrating digital storytelling into special education curricula and highlight avenues for future research in this field.

## Introduction

Early numeracy skills, also known as early numerical perception or early numerical knowledge, lay the foundation for developing advanced mathematical knowledge and play a critical role in an individual’s future mathematical success [1; 2]. These skills cover various aspects such as numeracy recognition, counting, numeracy patterns, numeracy comparison, numeracy operations, and estimation [2; 3]. Research has consistently shown that early childhood math skills can predict later reading and math achievement [1; 4]. With a solid foundation in early mathematics perception, students can learn and use mathematical concepts later [3; 5].

While some children acquire early numeracy skills instinctively [6], others require specific interventions to acquire these skills [7; 8]. Children with intellectual disabilities demonstrate delayed progress compared to their typically developing peers in acquiring early numeracy skills [9; 10]. Recent studies have revealed that children with intellectual disabilities have limited numerical perception skills and progress less in this area than their typically developing peers when starting formal education [10; 11]. Intellectual disability is the condition of being below normal in terms of general intelligence functions, such as attention, perception, memory, and reasoning, in addition to the inadequacy seen in adaptive behaviors that occur in the developmental period due to various reasons before, during, and after birth. Intellectual disability classification levels generally include mild, moderate, and severe intellectual disability. Each level reflects the degree to which cognitive functioning and adaptive behaviors are affected. Individuals with mild intellectual disabilities in this study may face particular challenges in academic and social settings but are generally capable of developing basic life skills with appropriate support and interventions. The need for interventions to develop the early numerical abilities of individuals with intellectual disabilities is emphasized for several critical reasons that profoundly affect their success in education and life. Numerical abilities form the basis of mathematical problem solving and analytical thinking skills that contribute to the capacity of individuals to solve the practical problems they face in their daily lives [12–16]. Consequently, the absence of foundational early numeracy skills in children with intellectual disabilities may lead to escalating challenges in their learning process [12].

Interventions targeting early numeracy skills can be effective for children with intellectual disabilities [17–20]. For example, a study investigating the effects of systematic instruction and individualized adaptations in early arithmetic lessons for primary school students with intellectual disabilities showed that physical adaptations to materials successfully increased students’ access to, and progress by contributing to, their early arithmetic skill development [19]. Davenport and Johnston [18] evaluated the effectiveness of an intervention strategy consisting of creating opportunities, providing prompts, providing feedback, and reducing prompts when teaching arithmetic/math skills to preschool children with intellectual disabilities, and found positive results. Interventions are more effective in improving math performance, primarily when implemented early [20; 21]. Furthermore, research has shown that basic numeracy skills are needed to perform daily tasks and that delays in early numeracy skills can have long-lasting effects on the development of mathematical abilities [21]. Consequently, interventions to improve the early numeracy skills of children with intellectual disabilities are crucial to their educational and life success. These interventions can meet the specific needs of children with intellectual disabilities and provide them with the basic skills necessary for mathematical thinking and problem-solving. Educators and researchers can support the development of math skills in children with intellectual disabilities by implementing early intervention.

Recent studies have shown that digital interventions can be more effective than traditional methods in improving mathematics skills of children with intellectual disabilities [22; 23]. Customized educational software, tablet applications, and interactive games allow students to progress at their individual learning pace, leading to a deeper understanding of the concepts [24; 25]. The contribution of technology in this area is highlighted by its ability to provide real-time feedback to students and create personalized learning experiences that can be immediately adapted [26]. This is especially critical for children with special needs to be educated according to their specific needs and learning styles [27; 28]. Moreover, these technological tools increase motivation and make learning fun [22–24].

### Digital story, early mathematics, and intellectual disabilities

Mathematical concepts can be complex for students because of their abstract nature [29]. The teaching and learning processes are in a constant state of evolution. It is entering a new era to meet global society’s needs and transfer 21st-century skills into educational settings. This change becomes even more critical when focusing on the needs of children with intellectual disabilities to learn mathematics [30]. Intellectual disability can further complicate the understanding and conceptualization of mathematical concepts [17]. Recently, the adaptation of skills to educational settings has been the focus of education and technology integration. This integration has been supported by various studies and research [22; 31; 32]. Digital storytelling is an effective form of technology that is used in education. Digital stories are educational tools that enable analytical thinking, enrich multiple language tools, and combine a constructivist approach with individual flexibility [33].

Digital stories support the understanding and learning of mathematical concepts for children with intellectual disabilities [34]. The Council of Teachers of Mathematics [NCTM] advises teachers to facilitate students’ understanding and conceptualization of mathematical concepts rather than simply repeating rote procedures [35]. Visual and interactive materials attract the students’ attention and help them learn concepts more effectively. Especially for students with intellectual disabilities, presenting abstract concepts, such as mathematics, by making them concrete through visual and interactive materials helps them better understand and internalize these concepts [36; 37] Thus, the development process of students’ mathematics skills becomes more effective and permanent. In this context, when the effect of digital storytelling in mathematics teaching is examined, it is seen that this method can potentially increase the mathematics achievement of children with intellectual disabilities [38].

Research has indicated that the incorporation of digital storytelling into mathematics education has the potential to enhance the understanding of mathematical concepts, problem-solving skills, and mathematical literacy among children with intellectual disabilities [39; 40]. Notably, students have expressed a positive inclination toward the use of digital storytelling in mathematics lessons, expressing a desire for continued application in future lessons [38]. This underscores the importance of effectively utilizing this teaching strategy in children with intellectual disabilities. However, there is a need for more concerted efforts to fully leverage this potential and implement digital storytelling more widely in mathematics education [40].

In conclusion, while the use of digital storytelling in mathematics education for children with intellectual disabilities has proven effective in clarifying abstract mathematical concepts, boosting problem-solving skills, and enhancing mathematical literacy, it is crucial to acknowledge that there is still considerable potential for exploration [21; 32]. As a result, this study aims to contribute to research on an age group, specifically kindergarten-level children, which has often been overlooked in the literature. In doing so, our study sought to provide insights into the importance of interventions tailored to the education of children in this age group, thereby contributing to broader trends in the literature on mathematical interventions. As emphasized by Starčič et al. [40], additional research endeavors and the formulation of robust theoretical frameworks are indispensable for fully unlocking the inherent capabilities of this instructional strategy. Thus, the primary objective of this study was to delve into the impact of digital stories on the early mathematics skills of students with mild intellectual disabilities, contributing to the ongoing exploration and refinement of effective educational interventions in this domain.

The following research questions were designed for this purpose:

1. "What are the effects of digital story interventions on the early math skills of students with ID?

## Method

This study was designed according to one of the experimental research models: the pretest/posttest/follow-up test control group. The experimental design uncovers the cause-effect relationships of variables whose effects are measured following applications conducted under specific rules and conditions. In experimental designs with pretest-posttest control groups, both experimental and control groups were established, and measurements were taken in each group before and after the experiment [41]. Furthermore, given the challenges associated with completely random assignment in educational settings, a quasi-experimental design was employed [41]. This design entailed assigning students to groups based on predetermined criteria to enhance the internal validity of the research. In this study, children in the experimental group received a digital story intervention, whereas those in the control group continued with their regular educational curriculum. The students were assigned to the experimental and control groups using stratified sampling to ensure a gender balance. This method was chosen to mitigate the potential influence of gender-based differences on research outcomes by ensuring a balanced gender representation in both groups.

### Ethics and approval and consent to participate

In this study, the data collection process aimed to investigate the effects of digital story-based early mathematics education on students with mild intellectual disabilities. The process was conducted as follows:

The data collection for the study began on August 1, 2023, and was concluded after an eight-week period on October 17, 2023. Follow-up data were collected on a single day, three weeks after the end of the intervention period.

Written informed consent was obtained from the families of all the participating students prior to the commencement of the study. This ensured that they were fully informed about the study’s aims, procedures, and ethical considerations, and consented to their children’s participation.

Each student and their family were informed that coding would be used instead of their real names to maintain confidentiality, thereby encouraging sincere responses to the research questions.

Unique coding were assigned to each participant, and the names and schools of the students were kept confidential.

This study received ethical approval from the Social and Human Sciences Ethics Committee of Dicle University.

### Study group

Students with mild intellectual disabilities aged between 6 and 7 years were included in the study. The students received individual and group education support two days a week at a special education rehabilitation center affiliated with the Ministry of National Education. These students also attended full-time mainstream education at public schools. All students attended the same rehabilitation center. All the participants had similar socioeconomic backgrounds. Considering the employment status of fathers in most families, it was observed that all fathers were actively working. Most of the mothers were housewives. When education levels were analyzed, it was observed that the majority of fathers were university graduates, while the majority of mothers were high school graduates. Classes with teachers of similar age and at least five years of experience were selected to control for the teacher variable.

The initial prerequisites for the participants were as follows: [a] having a diagnosis of mild intellectual disability, [b] no additional disabilities other than intellectual disability, [c] no absenteeism problems at the rehabilitation center, and [d] having limited early knowledge with a score below 25% on the 3rd Edition of the Test of Early Mathematics Ability [TEMA-3; 42]. The experimental group consisted of eight male and seven female students. While forming the control group, eight male and seven female students with the same prerequisites as the experimental group were included to control for gender-based differences. The average age of the students was 7 years and 2 months. According to the WISC-R test, children’s intelligence scores ranged from 65 to 70. This study was conducted in a city in southeastern Turkey.

### Setting

The research was conducted in an environment where students in a rehabilitation center received individual education. In this environment, there are chairs, tables, and blackboards. To avoid distracting students, the items in the classroom were placed behind the students and the seating arrangement was adjusted accordingly.

### Implementation process

Before starting the intervention program, interviews were conducted with the students’ families to inform them and to obtain written permission. In addition, information about the students was collected by interviewing their teachers in mainstream classes and at the rehabilitation center. The researcher conducted the entire implementation process.

### Content creation

The following steps were followed during the development of digital math stories. First, short stories were prepared by three academicians specializing in special education, three academically active in Turkish language teaching, and two special education teachers. Care was taken to ensure that these short stories were similar to those in Turkish books and suitable for the students’ grade level. The evaluation team thoroughly analyzed these short stories in terms of language structure, sentence length, word length, syllable count, and comprehensibility. Each story in the study is, on average, 15 pages long, and the number of syllables in each written page varies between 50 and 90. The researcher identified individual needs, considering each student’s cognitive abilities, learning styles, and math skills. For example, if a student excelled in counting but struggled with geometric shapes, a digital story such as "The Geometric Journey of the Forest’ was selected to focus on the student’s specific challenge. This story better responded to individual needs by providing specific content to strengthen students’ counting skills as well as their understanding of geometric concepts. Assessments and feedback helped deliver an effective learning experience tailored to each student’s needs.

### Determining the subject

In meetings held with three special education academicians who are experts in the field of education, a mathematics academician, and experienced educators, the main issues to be addressed in developing early mathematics skills were determined. These topics include counting, basic geometric shapes, measuring objects and patterns, and simple addition and subtraction operations. These topics were also included in the mathematics programs for the children in the control group. Similarly, these children receive early mathematics education twice a week in rehabilitation centers.

### Creating a story

A draft of entertaining and instructive stories suitable for selected topics was created. In line with the pedagogical approaches and suggestions of academicians, the story topics are as follows.

**Fig 1:**
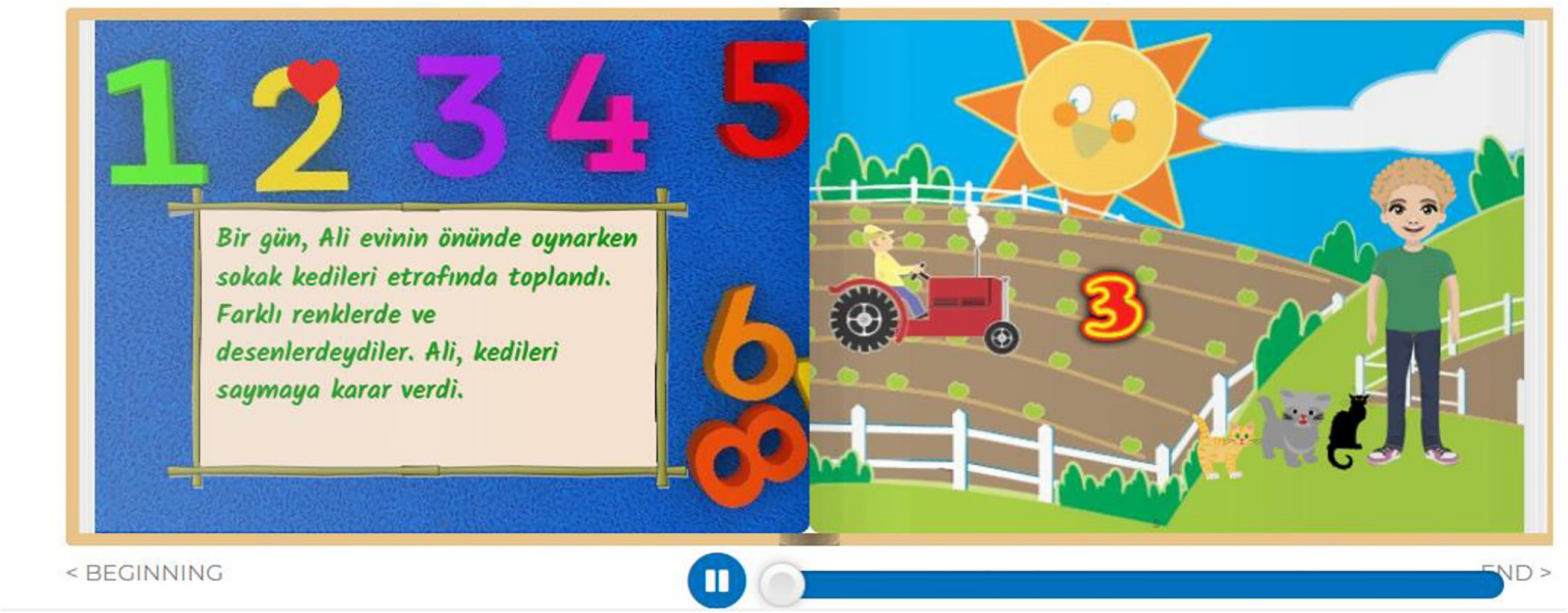
Sample visual from Ali’s Street Cats story.

*Ali’s Street Cats*: Ali counted the number of stray cats he encountered daily. One day, two cats; the other day, five cats… This counting adventure taught the students the basic concept of counting [see Fig 1].

**Fig 2:**
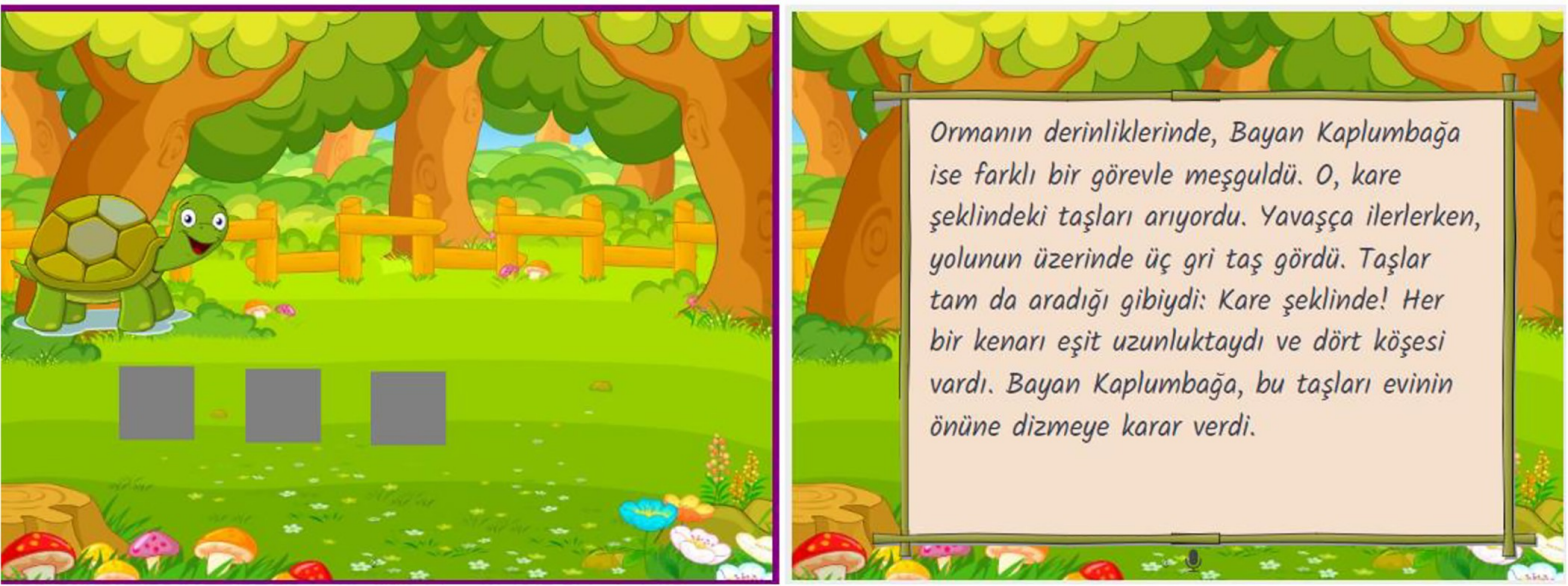
Sample visual from the story the Geometry Journey of the Forest.

*Geometric Journey of the Forest*: Animals pursue different geometric shapes in the forest. Mr. Rabbit had triangular leaves, and Miss Turtle found square stones. This journey introduced students to basic geometric shapes [see Fig 2].

*Mermaid’s Measuring Adventure*: While exploring treasures under the sea, Mermaid Marina needed to measure certain objects. She measured grains of sand with starfish and thus introduced students to the concept of measuring objects.

*Patterns of the Colorful Mountain*: Colorful birds living on top of the high mountain flew by, creating patterns in the sky. These patterns were designed to introduce students to simple pattern concepts.

*Math on the Farm*: Animals living on the farm have to collect or consume different amounts of food daily. On some days, they collected the feed; on other days, they removed the feed they ate. This story was designed for the students’ simple additions and subtractions.

The digital stories created included questions that focused on specific topics. These questions were added to evaluate the extent to which students with intellectual disabilities understood the skills and concepts of the story. Thanks to the questions in the stories, students were provided with instant feedback, thus supporting their understanding and learning processes.

#### Visual design

Digital stories were created using StoryJumper “https://www.storyjumper.com/” Appropriate images were selected from the StoryJumper library, or custom illustrations were uploaded. The story text was then added to the pages by visuals. In line with the suggestions of academics, colorful and eye-catching visuals were designed for each page to attract the children’s attention.

#### Voiceover

Each page was dubbed. In line with the recommendations of special education specialists, intonations and accents were used to attract the children’s attention.

#### Test phase

Digital stories were tested with a pilot group of students. This pilot study was conducted with ten students with normal development attending kindergarten. Student feedback was combined with observations of special education and mathematics academics, and the stories were revised accordingly.

#### Publication

After all revisions, the digital stories were uploaded to educational platforms and were ready for implementation.

### Pre-test applications

TEMA-3 is the only instrument in Turkey that measures general early mathematics skills, and validity and reliability studies have been conducted. Therefore, this method was preferred in the present study. At the beginning of the study, a pre-test was conducted to determine children’s current mathematical abilities. The Test of Early Mathematical Aptitude [TEMA-3] was used in the pre-test process. TEMA-3 was chosen to comprehensively measure children’s initial mathematical skills and knowledge. In addition, checklists were used during the pre-testing process to assess the children’s specific mathematical skills in more detail.

### Experimental group intervention process

Digital stories were prepared through the StoryJumper program, uploaded to educational platforms, and scheduled for implementation. The students’ instructional program was not disrupted by working two days a week for eight weeks. Each teaching session lasted 35 minutes on average. The intervention process for the digital stories was carried out personally by the researcher, a doctor of special education. The researcher has a relevant degree of specialization and experience in special education. During the intervention process, she identified student-specific approaches to assessing the level and needs of each student.

Initially, the level and needs of each student were assessed, and based on this assessment, the most appropriate digital story and supplementary material were identified. During the one-to-one sessions, the selected digital stories were presented to the students on a computer, and the mathematical topics in the story were emphasized. Throughout the training process, checklists were used to monitor the progress of early math skills. The 85 percent achievement criterion on these checklists assessed the students’ proficiency in each subject. After the 8-week intervention period, the educator initiated a follow-up process to assess the progress of students’ early math skills. This process aimed to measure the impact of digital stories by comparing students’ baseline and post-intervention levels.

### Control group implementation process

In the control group, it was pre-planned that the same mathematical topics would be emphasized as in the experimental group. However, the topics were presented to students using the direct instruction method. In this method, the teacher started by explaining the topic directly to the students and then practiced the topic with them. During the instruction, examples were presented for each topic, and students were asked questions based on these examples to check their level of understanding. Various additional activities and exercises were performed to help students better grasp the topic. This training process was conducted one-on-one by a special education teacher. The training continued for eight weeks, twice a week, each lasting 35 min.

### Final test process

After completing the training, a post-test was conducted to evaluate the acquired knowledge and skills. A general evaluation was conducted in the post-test using the TEMA-3 test. Simultaneously, checklists were used to examine the specific skills acquired by children in more detail. The checklists were designed to determine the extent to which children had acquired specific math skills and in which areas more support was needed. The results were used to compare performance before and after the intervention.

### Monitoring process

After completion of the training process, a monitoring process was planned to measure the long-term effects of the training. This follow-up was conducted three weeks after the end of the training. During this process, the children’s math abilities and skill gains were reassessed. The TEMA-3 test was administered again during the follow-up process, and checklists were used to identify specific skill gains.

### Social validity data

After the follow-up test, children and teachers were asked questions about the implementation, and their opinions were obtained.

#### Implementation reliability

Several strategies were implemented to ensure and assess implementation fidelity during the research process. These strategies aimed to ensure that the intervention was implemented as planned, aiming to maintain the consistency and accuracy of the intervention. Before the intervention began, the researcher conducted a comprehensive individualized assessment process. This assessment aimed to identify each student’s academic level, learning style, and needs. Based on these assessments, digital stories and supporting materials were selected to ensure a personalized approach in line with the main objectives of the intervention.

Throughout the eight-week intervention period, continuous monitoring of the implementation of the intervention and receiving feedback was carried out. This process aimed to contribute to the constant improvement of the intervention and strengthen the realization of the intervention by its purpose. A video camera was used to collect research data and record reliability data. Checklists containing critical steps identified in each intervention session were used. These lists were used as references for video evaluation by an independent researcher. The recorded sessions were evaluated according to these lists, and the degree to which the intervention was implemented as planned was quantitatively determined.

An independent researcher not directly involved in the intervention evaluated the recorded video sessions to ensure implementation reliability. This external evaluator systematically analyzed each session using the established checklists and provided an unbiased perspective on how well the intervention adhered to the selected protocols. At the end of the evaluations, based on the data obtained from all students, the reliability of the intervention was calculated to be 85%.

### Data collection tools

In this study, a "General Information Form" was used to collect the demographic and general information of the participants. In addition, the "Early Mathematics Ability Test [TEMA-3]" was used to measure children’s early mathematics ability. In order to make the evaluation process more systematic, the researcher prepared "Checklists" to monitor the student’s progress and evaluated them in line with the success criteria. After the implementation, feedback was obtained from children and teachers with the "Social Validity Form" developed by the researcher to evaluate the effectiveness and applicability of the education program. This form was used to determine general acceptance of the education program and possible difficulties in the implementation process.

### TEMA -3 [42]

The Test of Early Mathematics Ability [TEMA-3; 42] assesses the early mathematics abilities of children aged to 3–8 years. After its first version in 1983, the test was reintroduced in 1990 and 2003 in updated versions. TEMA-3 consists of 72 questions that measure early mathematics skills, focusing on concepts such as counting, writing, and establishing relationships between numeracy. There were also two separate test forms [Form A and Form B].

An adaptation and validity-reliability study in Turkey was conducted by Erdoğan [43]. In this adaptation process, the opinions of five experts in the field of child development and education were used to evaluate whether the items were appropriate for Turkish culture and the target age group. In line with the expert feedback, a Turkish version of the test was created. As part of the reliability study, both test forms were administered to 60-72-month-old children and the time-dependent consistency of the scale was evaluated by calculating the test-retest correlation. In the TEMA-3, the numeracy of correct answers constituted the raw score. These raw scores were converted into age equivalents, percentile ranks, and standard scores. In the original test, a standard score of 69 and below is considered "very poor," 70–84 "below average," 85–92 "average," 93–107 "above average," 108–115 "superior," and 116 and above "very superior" mathematics ability. However, the norming of this test has not been conducted in Turkey.

### Checklist

Within this study’s scope, a checklist was developed to assess students’ early mathematics skills. The checklist is based on five main topics with several items and questions for each topic. To develop the checklist, a literature review was conducted, interviews were held with experts in the field, and the validity of the checklist was tested through pilot studies. Under the topic of Numeracy Counting, the students’ ability to count the numeracy from 1 to 10 in the correct order, count backward from 10, and identify the missing numeracy among a given set of numeracy are questioned. This category had five questions in total, and 85% were accepted as the success criterion.

*In Basic Geometric Shapes*, students can recognize basic geometric shapes [e.g., triangle, square, and circle] and distinguish these shapes in different contexts. This section has six questions, and its success is expected to be greater than 85%.

*The Measurement with Objects category* focuses on the student’s ability to compare and sort objects by size, weight, and volume. The four questions in this category assess how students perceive the physical properties of objects and how they use these properties; again, the success criterion was set at 85%.

*In Patterns*, the focus was on the students’ ability to identify and maintain simple color, shape, or numeracy patterns. The students’ ability to identify the missing element in a sequence or to complete a pattern was tested using five questions in this section. The student’s achievement was targeted at 85% and above in this category.

*In the Basic Arithmetic Skill* subsection, the term "simple addition" refers to the fundamental arithmetic operation of combining two single-digit numbers without involving regrouping or carrying. The evaluation focused on students’ ability to perform basic addition operations correctly within a given set of numeracy. The six questions in this section required students to add pairs of single-digit numbers to assess their proficiency in this foundational aspect of arithmetic. The success criterion for this category was set at a rate of more than 85%, ensuring a comprehensive evaluation of the students’ competence in simple addition without the complexity of regrouping.

### Social validity form

Within the scope of this study, a series of steps were followed to design a social validity form for teachers and children. First, similar studies were examined through interviews with expert educators and a literature review. In line with the determined objectives, a five-question questionnaire was prepared for teachers. In this form, teachers were asked whether they were satisfied with the teaching of early mathematics skills, whether the program contributed to the development of children’s mathematical skills, whether they would like to implement this program in their classrooms, whether there was any aspect of the program they wanted to change, and whether they saw a change in children’s interest and motivation towards early mathematics skills.

A separate form consisting of three questions was designed for the children. This form was developed to measure whether the activities were fun, whether the students would like to see similar activities in their classrooms, and what they liked or disliked most about them. Both forms were carefully constructed to reflect the views of teachers and students more effectively and used clear language, which made it easy for the participants to answer the questionnaires. Questionnaires were administered in conjunction with interviews with the students and teachers. The questions posed to the students and teachers were carefully selected to allow participants to express their thoughts in depth. The researcher conducted the interviews one-on-one, and the answers to the questions were recorded.

### Data analysis

SPSS statistical program was used for data analysis. Due to the small sample size, nonparametric statistical tests were preferred [44]. Wilcoxon Signed Rank Test was used to determine the differences between pre-test and post-test scores. Mann-Whitney U Test was preferred to examine the score differences between different groups. In addition, effect sizes were also examined in group comparisons. The effect size was calculated by dividing the z value for Mann-Whitney U and Wilcoxon Signed Test by the square root of the sample size [45]. Cohen’s criteria [.1 = small, .3 = medium, and .7 = large] indicates the effect size [46].

## Results

The data obtained were analyzed in detail after completing the training and monitoring processes. The main findings of these analyses are presented below. The Mann-Whitney U-test was used to analyze whether there was a significant difference between the pre-test mean scores of the children in the experimental and control groups on the TEMA-3 and Control lists. The related results are presented in Table 1.

**Table 1:** Pre-test results regarding the scores of the children participating in the study in TEMA-3 and checklists.

	Group	N	$\bar{X}$	*SS*	Rank Mean	Rank Sum	*U*	*p*
**TEMA-3**	**Experiment**	15	84.60	7.89	16.07	241	104	.74
	**Control**	15	83.20	8.96	14.93	224		
**Numeracy Counting**	**Experiment**	15	1.33	0.48	15.00	225	105	.71
	**Control**	15	1.40	0.50	16.00	240		
**Basic Geometric Shapes**	**Experiment**	15	1.33	0.48	15.50	232	112	1.00
	**Control**	15	1.33	0.48	15.50	232		
**Measuring with Objects**	**Experiment**	15	1.00	0.75	16.23	323	101	.61
	**Control**	15	0.86	0.63	14.77	323		
**Patterns**	**Experiment**	15	1.33	0.48	15.50	232	112	1.00
	**Control**	15	1.33	0.48	15.50	232		
**Simple Addition and Subtraction**	**Experiment**	15	1.26	0.45	17.97	269	75	.65
	**Control**	15	0.86	0.63	13.03	195		

When Table 1 is examined, it is seen that the TEMA-3 pre-test scores of the children in the experimental and control groups were close to each other. The Mann-Whitney U-Test found no significant difference between the TEMA-3 pre-test mean scores of the control and experimental groups [U = 104, p = .74]. Similar results were found for the checklists. These results show that children are similar in terms of early math skills.

The Mann-Whitney U-Test results are presented in Table 2 to examine whether there is a significant difference between the post-test mean scores of the children in the experimental and control groups on the TEMA-3 and Control lists.

**Table 2:** Post-test results regarding the scores of the children participating in the study in TEMA-3 and checklists.

	Group	N	$\bar{X}$	*SS*	Rank Mean	Rank Sum.	*U*	*p*	*Effect*
**TEMA-3**	**Experiment**	15	90.46	2.64	21.33	320	25	.00	0.82
	**Control**	15	80.66	7.57	9.67	145			
**Numeracy Counting**	**Experiment**	15	4.73	0.45	23.00	345	.00	.00	0.89
	**Control**	15	1.53	0.51	8.00	120			
**Basic Geometric Shapes**	**Experiment**	15	5.73	0.45	23.00	345	.00	00	0.90
	**Control**	15	1.60	0.50	8.00	120			
**Measuring with Objects**	**Experiment**	15	4.00	0.00	23.00	345	.00	00	0.93
	**Control**	15	2.00	0.65	8.00	120			
**Patterns**	**Experiment**	15	4.60	0.63	22.90	343	1.50	00	0.89
	**Control**	15	2.20	0.41	8.10	121			
**Simple Addition and Subtraction**	**Experiment**	15	5.60	0.63	22.93	344	1.00	00	0.86
	**Control**	15	2.60	1.09	8.07	121			

Table 2 shows that the post-test scores of the children in the experimental group were significantly higher than those of the control group for TEMA-3, Counting, Basic Geometric Shapes, Measurement with Objects, Patterns, and Simple Addition and Subtraction Operations. In the TEMA-3 test, the average score of the experimental group was 90.46, while that of the control group was 80.66 [U = 25, p<0.01]. The effect size value also showed a large difference [0.82]. Similarly, significant differences and large effect sizes were observed in the control-list results.

The Wilcoxon signed-rank test was used to examine whether there was a significant difference between the pre-test and post-test mean scores of the experimental group children in the study regarding TEMA-3 and the control lists. The results are summarized in Table 3.

**Table 3:** Comparison of experimental group children’s pre-test and post-test score averages for TEMA-3 and control lists.

	Post -Test and Pre -Test	N	Rank Mean	Rank Sum.	*z*	*p*	*Effect*
**TEMA-3**	Negative Sequence	1	1 7	1 77	-2.98	.00	.76
	Positive Sequence	11					
	Equal	3					
**Numeracy Counting**	Negative Sequence	0	0	0	-3.49	.00	.90
	Positive Sequence	15	8	120			
	Equal	0					
**Basic Geometric Shapes**	Negative Sequence	0	0	0	-3.46	.00	.89
	Positive Sequence	15	8	120			
	Equal	0					
**Measuring with Objects**	Negative Sequence	0	0	0	-3.46	.00	.89
	Positive Sequence	15	8	120			
	Equal	0					
**Patterns**	Negative Sequence	0	0	0	-3.46	.00	.89
	Positive Sequence	15	8	120			
	Equal	0					
**Simple Addition and Subtraction**	Negative Sequence	0	0	0	-3.48	.00	.89
	Positive Sequence	15	8	120			
	Equal	0					

According to the Wilcoxon signed-rank test results in Table 3, the performance of the children in the experimental group on math concepts increased significantly after the intervention. The z-values obtained in TEMA-3, Counting, Basic Geometric Shapes, Measurement with Objects, Patterns, and Simple Addition and Subtraction, confirm this significant increase. All of these results have p < .001 and are statistically significant. Moreover, the effect sizes ranged from 0.76 to 0.90, indicating that the intervention had a significant effect. These findings suggest that the early math skills of children in the experimental group were positively supported after the intervention.

According to the results in Table 4, the experimental group scored higher than the control group on all criteria in both the post-test and follow-up tests. This shows that the training or intervention applied to the experimental group was effective. However, in some areas [e.g., "Measuring with Objects’], the effect size decreased slightly, which may indicate that the impact on these skills may diminish over time. Therefore, this type of training or intervention may need to be repeated or supported regularly.

**Table 4:** Follow-up test results for the scores of the children participating in the study in TEMA-3 and checklists.

	Group	N	$\bar{X}$	*SS*	Rank Mean	Rank Sum.	*U*	*p*	*Effect*
**TEMA-3**	**Experiment**	15	90.35	2.53	21.07	316	29.00	.00	.66
	**Control**	15	79.33	7.98	9.93	149			
**Numeracy Counting**	**Experiment**	15	4.73	.457	23.00	345	.00	.00	.89
	**Control**	15	1.53	.51	8.00	120			
**Basic Geometric Shapes**	**Experiment**	15	5.60	.50	21.80	327	3.00	.00	.84
	**Control**	15	1.78	1.05	7.71	108			
**Measuring with Objects**	**Experiment**	15	3.66	.81	21.37	320	24.50	.00	.70
	**Control**	15	2.13	.83	9.63	144			
**Patterns**	**Experiment**	15	4.40	.73	22.50	337	7.50	.00	.83
	**Control**	15	2.33	.61	8.50	127			
**Simple Addition and Subtraction**	**Experiment**	15	5.33	.81	22.80	342	3.00	.00	.85
	**Control**	15	1.93	1.16	8.20	123			

### Social validity findings

After the completion of the study, the "Social Validity Data Collection Form" was used to evaluate the effectiveness and functionality of the program in terms of children and teachers. Teachers answered "yes" to all of the questions except "Are there any aspects of the program that you would like to change?" and gave them positive opinions. The teachers stated that the children wanted more math activities in the classroom after the implementation, that the children waited impatiently for the implementation time, and that the implementation improved their math skills. For example, the first participant teacher said,’ The students liked the application very much. They said they would like to do mathematics activities more often in the classroom." The second participant’s teacher said, "This program improved students’ mathematics skills. They wanted to engage in similar activities in their classes. I think this program was successful". All the experimental group children who participated in the study reported that they found the application fun and wanted to carry out the application activities in their classes. All the children stated that there was nothing they did not like the application. For example, one student said,’ The application was enjoyable. Mathematics lessons are more interesting now, " and another student said, "There was nothing we did not like about the application. We liked it all very much.”

## Discussions

This study examined the effects of digital stories on the development of early mathematics skills in children with mild intellectual disabilities. The results of this study suggest that digital story-based education can be an effective method for improving the early mathematical skills of children with mild intellectual disabilities.

Incorporating digital storytelling into the curriculum for children with mild intellectual disabilities is a promising way to improve their mathematical abilities. The findings of this study support a growing body of research demonstrating the positive impact of technology-based interventions in academic education, especially for students with special needs [40; 47; 48]. Consistent results from various studies show that visual, interactive, and technology-based teaching approaches not only address the learning preferences of children with intellectual disabilities, but also increase their engagement [47; 48]. Digital storytelling enables the creation of multiple modes of representation and the sharing of interactive content that can facilitate narration and encourage students to actively participate in the learning process [40].

Active engagement is essential for students with intellectual disabilities because it can help them better understand mathematical concepts [49]. As Brown and Green [50] noted, this level of engagement increases students’ active participation in their learning journey, making the learning process more meaningful. Given that stories are inherently engaging and can be adapted to meet students’ learning needs when combined with digital media, it seems inevitable that intervention will be effective. The role of technological tools in education is becoming increasingly evident, especially in the preparation of educational materials for students with intellectual disabilities [48]. Özgüç and Cavkaytar [51] and Sherif and Boon [52] found that these tools increased the academic achievement of students with intellectual disabilities. Technology manifests itself not only as a way of presenting information to students, but also as a means of providing materials that suit their learning styles and needs. Such individually focused approaches can significantly facilitate the comprehension and internalization of content, especially for students with intellectual disabilities [47; 48; 52]. Therefore, all of these reasons are seen as the main factors behind the success of the intervention.

The results of the Social Validity Form provided essential data on how teachers and students perceived the intervention. When the results of the form for teachers were examined, it was observed that most teachers were satisfied with teaching early mathematics skills and believed that the program contributed to the development of children’s mathematical skills. In addition, most teachers expressed a desire to implement the program in their classrooms. This finding indicates that the program is valuable and effective. As for the results of the form for the students, most of the children stated that the program was fun and that they would like such activities in their classrooms. This shows that digital story-based interventions are educational, motivating, and engaging, as stated in related literature [22; 31; 32].

## Recommendations and limitations

This study examines the effectiveness of digital storytelling in children with mild intellectual disabilities. However, because a limited sample and specific learning context were used, the generalizability of the results may be limited. Therefore, studies involving groups of students with different levels of disability and learning styles are required. There may need to be more than the eight-week intervention period used in this study to evaluate the long-term effects of digital storytelling. Studies with more extended follow-up periods may help better understand the limitations of the sustainability of this method.

In parallel with the strategies implemented in this study, enriched teaching environments should be created for students with mild intellectual disabilities in other subject areas. Integrating digital stories into lessons and interactive questions related to these stories can increase students’ interest and participation in lessons. Although the study showed that digital storytelling is effective, there needs to be more comparison of this method with other teaching strategies, which should be considered a limitation. Therefore, future research should focus on comparative studies of different teaching strategies.

In cases where classroom education is restricted to global pandemics, such as COVID-19, it is essential to develop web-based, curriculum-integrated, and gamified platforms to ensure the continuity of the education process at home. The digital materials created in this study can be effectively used in the classroom, at home, or in different environments with family members. It is also essential to include such programs in teacher training programs and to train teachers on how to use this method more effectively. Special education and educational technology specialists should engage in collaborative projects to enrich special education curricula by using gamification techniques. The created digital materials can be integrated into popular operating systems such as Android and iOS, and made available to a broad audience in online environments such as websites or Web 2.0.

In conclusion, this study showed that digital storytelling can effectively teach mathematics to children with intellectual disabilities. However, this method should be tested using larger sample groups and mathematics topics. It is also essential to examine the long-term effects of digital storytelling and to compare this method with other teaching strategies. In this way, more information can be obtained about how digital storytelling can be used more widely in mathematics education for children with intellectual disabilities.

## Supporting information

S1 File Data set. (XLSX)
